# Participating in health insurance and health improvements for the relatively poor population: A propensity score analysis

**DOI:** 10.3389/fpubh.2022.968009

**Published:** 2022-09-15

**Authors:** Bin Hou, Yuxin Wu, Siyi Huang

**Affiliations:** School of Public Administration, Fujian Normal University, Fuzhou, China

**Keywords:** New Rural Cooperative Medical Scheme, health status, rural relatively poor population, propensity score matching, influencing mechanism

## Abstract

This study examined the causal relationship between participation in the New Rural Cooperative Medical Scheme (NRCMS) and health status among relatively poor population in rural China. Data were obtained from the China Family Panel Studies (CFPS) conducted in 2018, which contained 4,507 samples. This study used propensity score matching (PSM) to examine the net effect of participation in the NRCMS on the health of the relatively poor population, and this effect was tested for equilibrium using nearest neighbor matching, radius matching, and kernel matching. This study showed that participation in the NRCMS has a significant and positive effect on the health status of the relatively poor population and the positive health effect may come from three channels, including the increased frequency of physical activity, the fact that an individual is more likely to seek medical care at a lower level of visit, and a plan to reduce health care expenditures.

## Introduction

It has been proven that health is an important factor influencing poverty reduction and impoverishment (1), and health insurance can alleviate poverty vulnerability under health shock (2). In China, the distribution structure of the relatively poor population is mainly concentrated in areas with poor natural resource endowment, poor economic conditions and deep poverty. The group distribution of the relatively poor population mainly includes disabled individuals, widowed and orphaned individuals, elderly individuals, and individuals with long-term poverty due to illness. Therefore, regardless of the distribution structure of the relatively poor population or the distribution of specific groups, health problems will become one of the main obstacles in solving relative poverty. Health is an important issue raised by human capital theory. Human capital theory clearly puts forward that health is an important part of human capital, and high-quality human capital is crucial for promoting economic development and improving individual welfare (3). Therefore, in recent years, health insurance, as an international issue, has attracted the attention of all countries. Many developing countries have health insurance programs that provide health insurance for individuals or families through subsidies from government funds (4–6). The main purpose of health insurance is to reduce the medical expenses of an individual or a family, reduce the risk of becoming poor due to major diseases and help to spread the health risks that may arise.

Therefore, to improve the farmers' health level, reduce the medical burden, and solve the problem of poverty due to illness, in July 2003, the State Council of China started the pilot work of the New Rural Cooperative Medical Scheme (NRCMS). The NRCMS is funded by individual payments, collective support and government subsidies, which are led and organized by the government. It is a medical mutual aid system for rural residents that focuses on the overall planning of serious diseases and the settlement of minor diseases (7). It is a voluntary program and the voluntary feature was adopted to overcome public mistrust in the government-run insurance program (8, 9). The survey showed a high enrolment rate of 92% in this voluntary insurance scheme (10). Based on fee-for-service and cost-sharing models, the NRCMS mainly applies to inpatient services and some medical outpatient services, aiming to reduce the burden of disease costs for the rural population (8, 11). By 2008, the NRCMS covered both inpatient and medical outpatient services in most counties (9). The per capita annual premium of the NRCMS gradually increased from US $4.88 in 2005 to US $65.11 by the end of 2014 (12). By 2015, its coverage rate had reached 98 percent (13). At the same time, per-capita reimbursement caps have been raised, with outpatient reimbursement rising to $14, outpatient observation reimbursement to $163, and total medical expense reimbursement to $32,558 by the end of 2014. For hospitalization, 85 percent of the total expenses can be reimbursed by township hospitals, 70 percent by county hospitals, 55 percent by municipal hospitals and 50 percent by provincial hospitals (14).

Previous studies have examined the impact of health insurance on health status but have come to different conclusions. Researchers have found that participation in the NRCMS can improve the health status of rural residents mainly by reducing the incidence of poverty caused by illness or returning to poverty due to illness. In terms of actual effects, the NRCMS has a better poverty alleviation effect compared with previous medical insurance systems (8, 15). In addition, some researchers measured health status by using self-rated health and the number of illnesses or injuries in the past 4 weeks and studied the impact of participation in the NRCMS on health status. These researchers found that participation in the NRCMS did not significantly improve the health level of the participants (16, 17). Other researchers have studied the effects of health insurance in other countries. Researchers believe that medical insurance for rural areas will play a positive role in poverty reduction (18). Medical insurance can improve residents' health and reduce poverty vulnerability caused by health shock (19), while public medical insurance and premium subsidies can reduce the incidence of health poverty by 1/3 (1). In addition, Vietnam's Poor Health Protection Fund (HCFP) has significantly reduced cash expenditures on health for the poor population (20). Bangladesh's micro-health insurance scheme has significantly increased household income and stability and reduced the probability of falling into poverty (18). Medicare support programs in the United States have greatly reduced income inequality and improved welfare for the poor population by providing government subsidies (21).

Therefore, this study took the relatively poor rural population in China as the research object to investigate whether participation in the NRCMS has effectively improved the health status of this population. If the answer was yes, then the mechanism through which participation in NRCMS improved the health status of this population was further analyzed. Previous studies have mainly used multiple linear regression models to explore the relationship between health insurance and health, which has endogenous problems. In addition, the causal effect of medical insurance on the health status of the insured population should not be clear. Therefore, this study used the propensity score matching (PSM) to construct a counterfactual framework to test the causal relationship between participation in the NRCMS and the health status of the relatively poor rural population in China and then addressed the problem of causal inference that cannot be solved by traditional regression analysis. In this study, PSM was adopted to balance the confounding variables that affect participants' behaviors, which could solve the self-selection problem of the sample to some extent, and the net effect of this policy was obtained, providing a mechanism for explaining the health promotion effect of medical insurance.

## Methods

### Data

This study used data from China Family Panel Studies (CFPS), a survey designed and conducted by the China Center for Social Surveys at Peking University. CFPS data samples are nationally representative, and the survey team constantly updates the survey methods to ensure that the survey results are authentic and reliable, ultimately forming a database including family member answers, individual self-answers and individual proxy answers. The data cover 25 provinces, municipalities and autonomous regions in China, giving a more comprehensive picture of the demographic, economic and social characteristics of the Chinese population. In this study, CFPS data from 2018 were used. Combined with the database indicators required by the study, family sample codes were used as matching indicators to merge the data from a horizontal perspective. The final data content included variables such as household size, household income and health expenditures at the household level, as well as variables such as education level, income level, work status, smoking status and level of medical care at the individual level.

The object of this study was the relatively poor rural population. International experiences of relative poverty screening generally take a certain percentage of the median income as the threshold standard. For example, researchers propose using 50% of the median income as the relative poverty line (22). However, this kind of relative poverty screening based on the income method has some limitations; it ignores the essence of utility economics that describe that relative poverty is a subjective feeling. This subjective feeling is an individual's assessment of whether he or she is poor, and this self-assessment standard is usually related to the reference group set by the individual himself or herself. Therefore, this study drew on lessons from previous studies on subjective relative poverty and selected the relatively poor population based on the answers to the subjective relative poverty question (23). This study first selected individuals in rural households from the total sample and then selected individuals in the relatively poor population according to the their answer to the question “What is your income in the local area?” Answers was ranked on a scale of one to five; the higher the number, the higher an individual's local income. This study selected the population with indicators of one and two as the relatively poor population. After deleting invalid samples and sorting out data, 4,507 valid samples were finally obtained in this study.

### Variables and measures

Table 1 presents a description of variable.

**Table 1:** Definition and descriptive statistics of various variables.

	**Variables**	**Variable definitions**
Dependent variable	Health status	Continuous variable, ranging from 1 to 5
Independent variable	NRCMS	Categorical variable, NRCMS participants = 1, non-NRCMS participants = 0
	Age	Continuous variable
	Sex	Categorical variable, 0 = female, 1 = male
	Education level	Continuous variable, average education years
Control variables	Marital status	Categorical variable, 0 = unmarried, 1 = married
	Working status	Categorical variable, 0 = no, 1 = yes
	Frequency of internet use	Continuous variable, ranging from 0 to 6
	Smoking status	Categorical variable, 0 = no, 1 = yes
	Alcohol status	Categorical variable, 0 = no, 1 = yes
	Time spent watching TV	Continuous variable, ranging from 0 to 50
	Frequency of physical activity	Continuous variable, ranging from 0 to 50
	The level of medical care at the point of the visit	Continuous variable, ranging from 1 to 5
	Time spent performing housework	Continuous variable, ranging from 0 to 12
	Family size	Continuous variable, ranging from 1 to 15
	Health care expenditures	Continuous variable
	Bank loan repayment status	Categorical variable, 0 = no, 1 = yes
	Family income level	Continuous variable, take the logarithm

The independent variable in this study was whether an individual participated in NRCMS. Based on the question “What medical insurance system have you participated in?” in the questionnaire, the individuals participating in the NRCMS were assigned a value of 1, and those participating in other medical insurance systems and those not participating in a medical insurance system were assigned a value of 0.

The dependent variable in this study was the health status of individuals in the rural relatively poor population. Personal health is generally measured from subjective perceptions, medical diagnoses and physical function. Self-reported health status is an individual's subjective judgment, in accordance with his or her own objective health to be able to fully reflect the multidimensional nature of the health and integrity. Using this index can comprehensively reflect residents in terms of their physical, psychological, cognitive and external environments with multidimensional information and can form comprehensive evaluation results of their health more accurately (24). Therefore, this study chose the question of “How do you think your health is?” to measure the self-reported health status of the sample, and the answer to this question had reordered values for “1 =” unhealthy, “2 =” general, “3 =” more healthy, “4 =” very healthy, and “5 =” very healthy; the higher the value, the more healthy the rural relative poverty population was.

In addition, according to previous research conclusions, age, sex, education level, marital status, working status, frequency of internet use, smoking status, alcohol status, time spent performing housework, time spent watching TV, family size, bank loan repayment status and family income level were selected as control variables.

In theory, health insurance can affect an adult's health in three ways. First, under the financial incentive of medical insurance, residents may change their choice of medical institutions at different levels when seeking medical services, which may ultimately affect their health status (25). Second, after participating in medical insurance, people are more likely to choose more diversified physical exercise methods and increase the frequency of physical activity, thus affecting their health status (26). Finally, health insurance can also influence the health status of the population by reducing incentives to take precautions against personal health, such as reducing the purchase and use of health supplies (27). Therefore, we chose the level of medical care at the point of the visit, the frequency of physical activity, and health care expenditures as important mechanism of action variables to examine. Among them, the measurement question corresponding to the variable of the level of “medical care at the point of the visit is” where do you usually go if you want to see a doctor, “and the corresponding option is” 1 = general hospital, 2 = specialized hospital, 3 = township health center, 4 = village health room, 5 = clinic. “The answer is reversely coded, indicating that the higher the value is, the higher the level of medical care is. The variable of the frequency of physical activity corresponds to the measurement question” How many times do you get physical activity in the past week. The forms of physical activity include different forms of outdoor activities, all kinds of ball games and contact sports. The variable of the health care expenditures corresponds to the measurement question “How much did you spend on health care in the last 12 months?”

### Propensity score matching

The PSM was proposed by Rosenbaum and Rubin (28), and it is considered that the propensity score is the conditional probability influenced by an explanatory variable under the control of numerous observable confounding variables. The propensity score matching method is a popular new statistical method for social science. This method allows researchers to focus on the independent variable intervention implementation randomized allocation, which can overcome the problem of choice; at the same time, it can maximize the limit for confounding variables and make the probability of an event occurring in the treatment group and the control group close to achieve the purpose of a simulation experiment. In addition, the role of propensity score matching lies in the construction of a “counterfactual framework”. The occurrence of an event that an individual experiences may depend on two opposite states. However, in real life, people can only be in one life state and cannot re-experience the other state. We can use PSM to construct a “counterfactual framework” to address the problem of causal inference that traditional regression analysis cannot solve. In traditional regression models, there are many confounding variables between explanatory variables and explained variables, so it is difficult to find the net effect between explanatory variables and explained variables. Therefore, the PSM method can put these confounding variables into the logit model to predict the propensity value and then control the propensity value to mitigate the biased causal inference caused by selection bias. Therefore, this study first established the OLS model as a benchmark model for the data as a whole to test the impact of participating in NRCMS on the health status of individuals in the relatively poor rural population. Then, on the basis of overcoming selection bias due to participating in the NRCMS, the conclusions of the OLS model were further verified to investigate whether such influence continues to exist through the establishment of the PSM method.

## Results

### Baseline regression results

In this study, a nested OLS model was used to empirically test the impact of participating in NRCMS on the health status of individuals in the relatively poor rural population. Table 2 shows the baseline results of the regression model. As seen in Table 2, the health level of NRCMS participants was 9.6% higher than that of non-NRCMS participants, and the test result was significant at the 5% level.

**Table 2:** OLS regression results of the health level of the relatively poor population in rural areas.

**Comparison term**	**Health status**
**NRCMS (non-NRCMS participants)**	
NRCMS participants	0.096^**^
	(0.039)
Control variables	All control variables
Constant term	1.382^***^
	(0.282)
N	4,507
F	22.96^***^
*R* ^2^	0.080

*P < 0.10,

** P <0.05,

*** P < 0.01;

The value in brackets is standard error.

### Selection bias of participation in the NRCMS

Table 3 shows the results of the chi-square test or *T*-test for participation in the NRCMS and confounding variables. The preliminary results of the chi-square test or *T*-test showed that selection bias in the samples obtained by the NRCMS did exist, and the results of other confounding variables showed a significant correlation at the 1% level, except for the test results of sex and smoking status at the 10% level. This showed that the distribution of the samples for participation in the NRCMS was unbalanced. Due to the existence of these selectivity biases, processing allocation is unavoidable and therefore needs to be further corrected.

**Table 3:** The result of selection bias of participation in the NRCMS.

**Comparison term**	**Percentage of NRCMS OR Mean of NRCMS vs. non-NRCMS**	**χ^2^ or *t*-test**
Age	33.442 (3,478) vs. 30.859 (1029)	0.000
Sex	25.76(896)	0.072
	74.24 (25,82)	
Education level	8.545 (3,478) vs. 11.087(1029)	0.000
Marital status	85.42 (2,971)	0.000
	14.58 (507)	
Working status	90.86 (3,160)	0.000
	9.14 (318)	
Frequency of internet use	4.980 (3,478) vs. 5.328 (1029)	0.000
Smoking status	4.49 (156)	0.053
	95.51 (3,322)	
Alcohol status	6.38 (222)	0.000
	93.62 (3,256)	
Time spent watching TV	9.194 (3,478) vs. 8.204 (1029)	0.000
Frequency of physical activity	1.801 (3,478) vs. 1.780 (1029)	0.000
The level of medical care at the point of the visit	3.304 (3,478) vs. 3.400 (1029)	0.000
Time spent performing housework	1.903 (3,478) vs. 1.152 (1029)	0.000
Family size	5.772 (3,478) vs. 4.906 (1029)	0.000
Health care expenditures	179.309 (3,478) vs. 365.761 (1029)	0.000
Bank loan repayment status	15.12 (526)	0.000
	84.88 (2,952)	
Family income level	10.944 (3,478) vs. 11.199 (1029)	0.000
Health status	3.255 (3,478) vs. 3.143 (1029)	0.000

### Propensity score prediction

In this study, all confounding variables were simultaneously incorporated into the binomial logit regression model to predict the probability of the influencing samples' participation in the NRCMS. Table 4 shows the results of the regression model. Education level, marital status, working status, smoking status, alcohol status, time spent watching TV, time spent performing housework, family size, bank loan repayment status, family income level and other variables have a significant impact on the probability of participating in the NRCMS. Therefore, the selective problem of whether individuals participate in the NRCMS objectively exists; that is, these confounding variables can significantly predict whether individuals participate in the NRCMS.

**Table 4:** Binomial logit propensity prediction model for rural relatively poor population to participate in NRCMS.

**Control variables**	**NRCMS**
Age	−0.098 (0.007)
Sex	0.032 (0.099)
Education level	−0.131*** (0.012)
Marital status	0.576*** (0.113)
Working status	−0.678*** (0.167)
Frequency of internet use	−0.042 (0.027)
Smoking status	0.647*** (0.22)
Alcohol status	1.077*** (0.207)
Time spent watching TV	0.028*** (0.005)
Frequency of physical activity	0.012 (0.012)
The level of medical care at the point of the visit	−0.136*** (0.047)
Time spent performing housework	0.273*** (0.034)
Family size	0.162*** (0.019)
Health care expenditures	−0.0004 (0.0003)
A bank loan was taken out	0.808***(0.129)
Family income level	−0.461***(0.063)
Chi-square	745.39^***^
−2 Loglikelihood	4097.125
Pseudo *R*^2^	0.1539

### Propensity score matching

#### Matching quality test: Presentation of the common range of propensity scores

**Figure 1:**
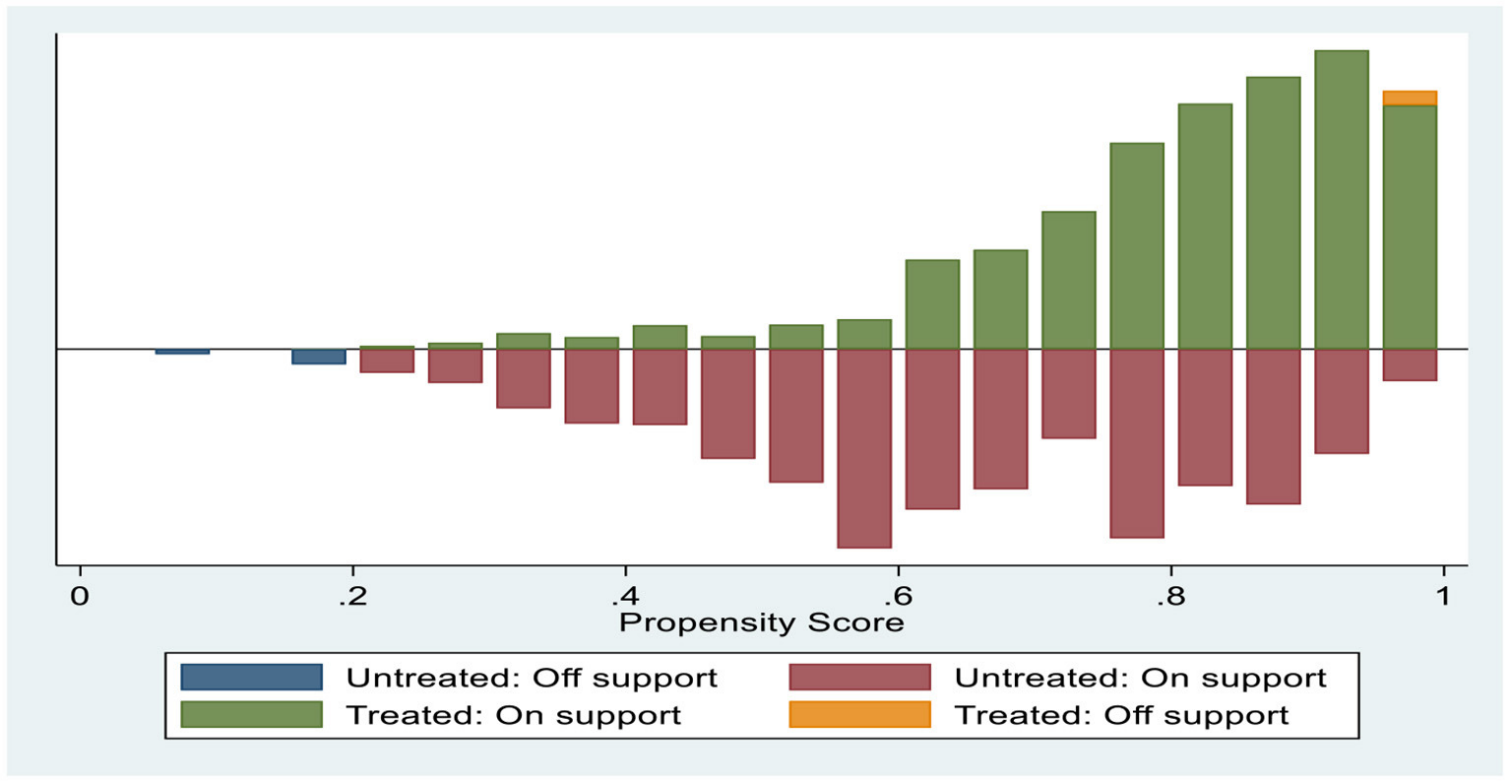
Common range of values for propensity score.

Figure 1 shows the common range of the propensity scores. Propensity matching requires that the distribution of propensity values overlaps greatly between the control group and the treatment group. As seen in Figure 1, the common support interval between the group that participated in the NRCMS and the group that did not participate in the NRCMS was relatively large, which means that the data application conditions were good.

Table 5 shows the common range of supported domains for propensity score matching. As seen in Table 5, among the 4,507 observed values, 12 values in the control group were not in the common value range, 29 values in the treatment group were not in the common value range, and the remaining 4,466 values were in the common value range.

**Table 5:** The common range of supported domains.

**Treatment assignment**	**Off support**	**On support**	**Total**
Untreated	12	1,017	1,029
Treated	29	3,449	3,478
Total	41	4,466	4,507

#### Balance test of the sample

Table 6 shows the balance test results of the samples. The results showed that no matter which preference matching method was used, all variables were almost balanced in the NRCMS participants group and the non-NRCMS participants group. After matching, the differences of all variables between the two groups of samples were no longer significant, which indicates that propensity score matching achieved sample equilibrium results to a certain extent.

**Table 6:** Comparison of sample characteristics before and after matching.

**Variable**	**Sample**	**Mean value**		**Standardized bias (%)**	**Bias reduction (%)**	**T Value**	***P*-Value**
		**Treated**	**Control**				
Age	Unmatched	33.442	30.859	35.5		9.70	0.000
	Matched	33.416	33.289	1.7	95.1	0.73	0.464
Gender	Unmatched	0.258	0.286	−6.3		−1.80	0.073
	Matched	0.260	0.228	3.1	23.7	0.17	0.224
Education	Unmatched	8.545	11.087	−63.9		−18.57	0.000
	Matched	8.603	8.335	1.6	89.4	0.69	0.787
Marital status	Unmatched	0.854	0.703	37.1		11.26	0.000
	Matched	0.853	0.850	0.6	98.5	0.27	0.786
Working status	Unmatched	0.909	0.951	−16.8		−4.42	0.000
	Matched	0.909	0.853	4.1	−30.2	0.18	0.135
Internet frequency	Unmatched	4.980	5.329	−21.5		−5.78	0.000
	Matched	4.981	4.636	3.9	45.8	0.53	0.712
Smoking status	Unmatched	0.045	0.031	7.2		1.94	0.053
	Matched	0.045	0.026	4.2	−39.4	4.29	0.000
Alcohol status	Unmatched	0.064	0.033	14.4		3.75	0.000
	Matched	0.064	0.065	−0.3	98.1	−0.10	0.922
Housework time	Unmatched	1.903	1.152	49.1		12.89	0.000
	Matched	1.850	2.230	−2.1	40.6	−0.26	0.309
Time spent watching TV	Unmatched	9.194	8.204	12.9		3.58	0.000
	Matched	9.136	9.405	−3.5	72.8	−1.32	0.186
Family size	Unmatched	5.772	4.906	38.1		10.36	0.000
	Matched	5.772	4.444	3.5	−53.3	0.45	0.576
Bank loan repayment	Unmatched	0.151	0.089	19.1		5.08	0.000
	Matched	0.148	0.133	4.7	75.6	1.80	0.171
Family income	Unmatched	10.944	11.199	−36.4		−10.12	0.000
	Matched	10.953	10.876	11.0	69.7	4.56	0.000

#### Propensity score matching results

There are two results of propensity score matching, namely, the ATT (average effect of health status of actual NRCMS participants) and ATE (average effect of health status of NRCMS participants and non-NRCMS participants). This study focused on the impact of participation in NRCMS on health status, so the ATT needed to be observed. Table 7 shows the ATT obtained by using various matching methods, including in 1-to-1 matching, 1-to-4 matching, radius matching and kernel matching. The T value of the ATT corresponding to 1-to-1 matching, 1-to-4 matching, radius matching and kernel matching in the nearest neighbor matching method were 4.17, 4.21, 4.85, and 3.5, respectively, which were all greater than the T value corresponding to a 1% significance level of 2.76, indicating that after controlling for sample selectivity bias, participation in the NRCMS has a positive effect on the health level of the participants. In other words, participation in the NRCMS can improve the health of individuals in the relatively poor rural population.

**Table 7:** PSM results.

	**Health status**		
	**ATT**	**SE**	**T Value**
1 to 1 neighbor matching	0.327	0.078	4.17
1 to 4 neighbor matching	0.316	0.071	4.43
Radius matching	0.298	0.048	2.77
Kernel matching	0.271	0.049	3.35

### Impact mechanism analyses

We attempted to explore the impact mechanism behind the influence of participation in the NRCMS on health status. Table 8 presents the potential impact mechanisms of participation in NRCMS on health status. The results show that participation in NRCMS significantly increased the frequency of physical activity including different forms of outdoor activities, all kinds of ball games and contact sports at a different significance level. Studies have proved the relationship between health insurance and physical activity or outdoor activity in different groups. Using panel data from China Rural Mutual Health Care, the researchers analyzed the impact of medical insurance on the health status of enrollees and found that medical insurance had a positive impact on the daily physical activity of people over 55 years old (29). Besides, the researchers analyzed the impact of NRCMS on the health of the elderly in rural areas by using data from Chinese Longitudinal Health Longevity Survey, and found that the frequency of outdoor activities expressed by the elderly participants was higher than that of non-participants. NRCMS promoted the awareness of outdoor activities of the elderly and realized the goal of improving the physical function of the elderly (30). Furthermore, compared with people of higher and middle socioeconomic status, people of lower socioeconomic status may suffer worse physical health due to higher family social stress and lower stress control ability (31). In a randomized cross-sectional study of 1,650 adult men aged 20 to 50 years from rural Bangladesh, the researchers found that compared with men of middle and higher socioeconomic status, men of lower socioeconomic status with higher family socio-demographic stress, economic hardship, family stress and lower psychological resources are most likely to suffer from poor physical health (32). Therefore, the relatively poor population in rural areas will pay more attention to their own physical conditions, participation in NRCMS has a significant impact on the frequency of physical activity by providing affordable and cost-effective health care services and enhancing their awareness of health care (33). Regardless of the reimbursement or compensation granted to the relatively poor population, maintaining a healthy body and reducing the utilization of medical care remains the optimal solution. Increasing the frequency of physical activity is a direct and effective method.

**Table 8:** Impact mechanism analyses of NRCMS on health status.

	**The level of medical care at the point of visit**			**Frequency of** **physical activity**			**Health care expenditure**		
	**ATT**	**SE**	**T Value**	**ATT**	**SE**	**T Value**	**ATT**	**SE**	**T Value**
1 to 1 neighbor matching	−0.113	0.056	−1.77	0.168	0.181	1.93	−0.119	0.113	−1.82
1 to 4 neighbor matching	−0.171	0.051	−1.69	0.058	0.166	1.35	−0.176	0.214	−1.97
Radius matching	−0.188	0.037	−2.37	0.123	0.124	2.99	−0.182	0.147	−2.44
Kernel matching	−0.191	0.038	−2.39	0.105	0.126	1.83	−0.219	0.175	−2.76

In addition, we found that participation in NRCMS reduced the possibility of seeking medical care at a higher level of visit and taking health care expenditures at a different significance level. The above conclusion can be inferred that medical insurance may bring a series of negative effects, one of the important problems is the moral hazard which occurred to the insured. Moral hazard refers to the tendency of the insured to increase the consumption of medical services, which has been studied by a large number of theoretical and empirical work (34–36). The moral hazard can be divided into two kinds: ex-post moral hazard and ex-ante moral hazard. Ex-post moral hazard refers to the behavior of patients who overconsume medical resources after they have health insurance because they face lower marginal prices, such as extending hospital stay or choosing a lower level of hospital stay (37). Ex-ante moral hazard refers to that when patients participate in insurance, their motivation and activities of self-protection and disease prevention will decrease as the disease loss they face will be reduced due to the insurance compensation (27). For example, the researcher found that medical insurance coverage poses a significant moral hazard to the probability of access to health care (38). In addition, in the case of lower income level, medical insurance will bring about implicit behavior or moral hazard incentive effect, and poor policyholders are more inclined to use hospital facilities and medical public services. The decline in the price of medical services due to insurance coverage increases utilization by the poor to a greater extent (39). Therefore, the above conclusions inspire us to prevent the possibility of moral hazard arising from participating in NRCMS.

Thus, these findings support the view that participation in NRCMS has a strong impact on the frequency of physical activity, the choice of the level of medical care and expenditure on health care, which in turn highlights possible mechanisms by which health insurance improves health outcomes.

## Discussion

### Participation in the NRCMS has a significant positive impact on the health status of individuals in the relatively poor rural population

In this study, we investigated the impact of participation in the NRCMS on the health status of individuals in the relatively poor rural population using CFPS 2018 data. In the process of investigation, we mainly used PSM to discover the net effect of participation in NRCMS on health status. The evidence showed that participation in NRCMS had a significant and positive impact on the health of relatively poor individuals in rural areas. This is consistent with the conclusions of previous studies. In a survey of 2,093 rural adults, the researchers found that NRCMS participation had a significantly positive impact on their self-assessment and mental health (40). In addition, the researchers used data from the 2013 China Health and Retirement Longitudinal Survey to study the impact of medical insurance on the health status and life satisfaction of elderly individuals, and the results showed that medical insurance contributes to the improvement of health status and life satisfaction of elderly individuals (41).

### Participation in the NRCMS influences health status by influencing the frequency of physical activity, the choice of the level of medical care and expenditure on health care

This study found several mechanisms by which participating in the NRCMS affects health. First, after participating in the NRCMS, relatively poor rural people will pay more attention to their physical condition, actively participate in daily exercise, and deliberately increase the amount of exercise, which will be more conducive to their health. Therefore, participating in more physical activity is a channel to improve health. Second, NRCMS participants improve their health by increasing their chances of seeking medical care at relatively low-quality medical institutions. As individuals in the relatively poor population mainly live in rural areas, the relatively low level of medical facilities can improve their immediate access to health care, thereby improving their health status. This mechanism may be related to the implementation of a hierarchical medical system. Third, NRCMS participants spend less on preventive health care, which in turn highlights possible mechanisms through which health status is changed with medical insurance.

### Implications

These findings have important policy implications. First, it is necessary to further expand the coverage of medical insurance and improve health status through comprehensive medical insurance coverage. It needs more strengthening by encouraging peoples' participation into NRCMS with a necessity to implement a new reimbursement payment system by health care providers (42). Second, the mechanism of medical insurance financing and compensation should be appropriately oriented to vulnerable groups and areas in difficult circumstances, especially to relatively poor groups, such as individuals with subsistence allowances, individuals who are extremely poor, individuals in low-income groups, families in poverty caused by illness and seriously ill patients. Third, in order to prevent the occurrence of moral hazard, medical insurance should combine with long-term care insurance, strengthen the propaganda of disease prevention and increase individual awareness of disease prevention. At the same time, the policy of providing a free medical check-up every year as a reward for those who do not use any of the reimbursed medical services can effectively prevent moral hazard and promote the use of preventive health care among participants.

The marginal contribution of this study is as follows: First, based on the data of a large national micro tracking survey, this study specifically studied the impact of health insurance on the health of the relatively poor population, providing new empirical evidence for existing relevant studies. Second, this study balanced the confounding variables that affect the behavior of insured individuals through PSM, which can solve the self-selection problem of a sample to a large extent; this was conducive to the net effect of the policy impact and improved the accuracy of the estimated results. Third, from the perspective of the influencing mechanism, this study analyzed the channels through which medical insurance affects the health level, which provides a reference value for the formulation of relevant policies.

### Limitations

This study has the following limitations. First, considering the differences in the reimbursement ratio of medical insurance in different regions, such differences will have different impacts on the health services and health status of insurance participants. However, due to the limitation of the data, we could not control this variable. Second, some other control variables, such as the urbanization index and air pollution index, are also important factors affecting the health status of a population. However, due to the limited data content, we could not control these variables. When more data are available, we will control these factors. Third, as the research object of this study is the relative poor population in rural areas and the relationship between their participation in the NRCMS and their health status, this study does not analyze the frequency of physical activity of the urban poor population after they participate in health insurance. However, the comparison of the two groups could be studied as a new topic in the future.

## Data availability statement

Publicly available datasets were analyzed in this study. This data can be found at: http://www.isss.pku.edu.cn/cfps/.

## Author contributions

BH was responsible for writing and contributed to the conception of the work, data analysis and interpretation, and drafting the article. SH and YW contributed to the data collection, data analysis and interpretation, and give important revising suggestions. All authors contributed to the article and approved the submitted version.

## Funding

This study was supported by Fuzhou Social Science Planning (2021FZB08) and Education and Scientific Research Project of Young and Middle-aged Teachers of Fujian Provincial Education Department (Social Science) (JAS19035) awarded to BH.

## Conflict of interest

The authors declare that the research was conducted in the absence of any commercial or financial relationships that could be construed as a potential conflict of interest.

## Publisher's note

All claims expressed in this article are solely those of the authors and do not necessarily represent those of their affiliated organizations, or those of the publisher, the editors and the reviewers. Any product that may be evaluated in this article, or claim that may be made by its manufacturer, is not guaranteed or endorsed by the publisher.
